# Local residents’ attitudes toward and contact with international students: a perspective from Montreal, Quebec

**DOI:** 10.3389/fpsyg.2024.1484985

**Published:** 2024-11-13

**Authors:** Oguzhan Tekin, Pavel Trofimovich

**Affiliations:** ^1^Institute for the Study of University Pedagogy, University of Toronto, Mississauga, ON, Canada; ^2^Department of Education, Concordia University, Montreal, QC, Canada

**Keywords:** international students, host communities, prejudice, integrated threat theory, linguistic threat, intergroup contact

## Abstract

As migrants holding temporary, foreign-resident status in their host communities, international students often experience prejudice and have little meaningful contact with locals. To date, a comprehensive account of international students’ experience is lacking, and existing conceptualizations exclude linguistic threat as a potential source of increased prejudice and diminished contact. Therefore, our goal in this study (set in Quebec, Canada) was to explore local residents’ attitudes toward and contact with international students in relation to five potential threats experienced by local residents, including cultural differences, competition over resources, intergroup anxiety, stereotypes, with linguistic threat added as a new, previously unexplored variable. We recruited 59 student and non-student local francophone residents as participants and examined their attitudes toward, perceptions of threat from, and the quality and quantity of contact with international students attending English-medium universities. Both student and non-student participants demonstrated positive attitudes toward and low levels of perceived threat from international students, except for linguistic threat. Compared to student participants, non-student participants reported significantly greater linguistic threat from international students and described contact with them that was both less frequent and lower in quality. Regression models accounted for 50–67% of variance in participants’ attitudes, with symbolic threat to social values and belief systems emerging as the common significant predictor of attitudes for both groups. Adding linguistic threat did not improve regression models. Finally, only contact quality showed significant relationships with attitudes and perceived threat, where greater contact quality was associated with more favorable attitudes and reduced threat. We discuss implications of intergroup attitudes and contact for language planning and use in multilingual contexts.

## 1 Introduction

Contact among ethnolinguistically and culturally diverse social groups has become more prominent than ever, and the continuously growing population of international students around the world is but one manifestation and a reminder of this reality. Between 2020 and 2021, 6.4 million students left their home countries to study abroad (International Organization for Migration, 2024), and the majority chose English-speaking countries such as the United States, Canada, the United Kingdom, and Australia (OECD, 2023). Considered a safe, stable, and tolerant country to study and live (CBIE, 2021), Canada ranked third among these countries regarding the number of international students in 2019 (El-Assal, 2020) and is currently hosting more than a million international students. Following Ontario and British Columbia, Quebec ranks third among Canadian provinces, with about 11% of these international students (CBIE, n.d.). However, international students, with their diverse ethnic, social, and linguistic backgrounds, often find it difficult to adapt and socially integrate into many host communities (Paradowski et al., 2021; Volet and Ang, 2012). Establishing contact with local residents seems to be particularly challenging (CBIE, 2015; Gresham and Clayton, 2011; Rajapaksa and Dundes, 2002), which has been generally attributed to prejudicial attitudes and discriminatory behaviors by the host community. Yet comprehensive investigations of potential sources of such attitudes and behaviors are conspicuously missing. Therefore, the present study aims to address this gap by examining potential sources of negativity and discrimination toward international students from members of a local host community and by exploring the link between such negativity and intergroup contact between these two groups.

## 2 Background literature

### 2.1 Prejudice against international students

International students have attracted much attention not only among researchers but also policymakers and institutions of higher education due to the socioeconomic benefits they bring to their host countries (Smith, 2016). Besides direct economic contributions to their host communities, for instance, in terms of considerable expenditure on food, accommodation, and transportation (Esses et al., 2018; Global Affairs Canada, 2020), international students also generate revenue for their host institutions paying three or four times more in tuition (Anderson, 2015), creating thousands of university jobs (El-Assal, 2020), and contributing billions of dollars in tax revenue (Global Affairs Canada, 2020). From a social perspective, international students bring a multitude of cultures, languages, and ways of thinking to their host countries and enable members of their respective host communities to connect with the outside world without having to travel (Anderson, 2015; CBIE, 2015). Given that only a small proportion of Canada-born students (3.1%) opt to study abroad (Internationalization in Canadian Universities, 2014), international students also bring major benefits to their Canadian peers in terms of cultivating intercultural awareness and communication skills.

Yet despite what they offer to their host communities, international students frequently report feeling unwelcomed and experience discrimination based on their race, skin color, ethnicity, and cultural practices (Briscoe et al., 2022; Gareis, 2012; Samuel and Burney, 2003; Tran, 2017), or due to language-related issues such as accented speech or low target-language proficiency (Gbadamosi, 2018; Lindemann, 2005; Maeda, 2017; Surtees, 2019). For instance, international students from Asia studying at a vocational school in Australia reported being singled out by the locals, who labeled them “PR hunters,” or individuals who exploit the system by arriving to study in Australia with the sole purpose of obtaining permanent residency (Tran, 2017). In the United States, international students reported being ridiculed for their language errors and accents, which caused them to feel embarrassed, socially isolated, and unwilling to participate in class activities (Maeda, 2017).

Considering that persons of color (e.g., students from India and China) constitute more than half of the international student population worldwide (OECD, 2023) and almost 75% of the international students in Canada (CBIE, n.d.), prejudice can indeed be a major issue for most international students (Poyrazli and Lopez, 2007), leading to the perception of “otherness” or outright discrimination and ultimately causing adaptation problems and subpar educational experiences. For instance, studying a large group of international students in the United States, with students from China being the majority, Wadsworth et al. (2008) showed that perceived discrimination negatively impacted students’ satisfaction with their study abroad. In another study, perceived discrimination also contributed to students’ feelings of social isolation and homesickness (Poyrazli and Lopez, 2007). Indeed, in their majority, international students tend to experience more social adjustment problems than their domestic peers (Hechanova-Alampay et al., 2002). However, because international students are a heterogeneous group with distinct experiences (Grayson, 2007), students from some countries of origin, particularly in Africa, Asia, and the Middle East, might find social and academic adjustment more difficult than other students because of overt and covert discrimination (Jean-Francois, 2019; Lee and Rice, 2007; McDonough et al., 2022; Senyshyn et al., 2000; Yeh and Inose, 2003).

Although most challenges faced by international students tend to be treated as issues to be dealt with by students themselves (Harryba et al., 2013), other challenges—and especially prejudicial and discriminatory attitudes and behaviors—must be addressed with respect to each host community (Lee and Rice, 2007). Most research thus far, however, has not comprehensively examined potential reasons that may trigger negativity from a host community toward international students, with different sources of prejudicial and discriminatory attitudes and behaviors targeted in separate investigations. Some studies, for instance, have implied that negative attitudes can arise from differences in cultural values (Bonazzo and Wong, 2007; Lee and Rice, 2007; Pritchard and Skinner, 2002) and perceived competition over limited resources such as university admission, employment, course grading (e.g., when grading is done on a curve), and attention from instructors (Barron, 2006; Charles-Toussaint and Crowson, 2010; Hanassab, 2006; Myburgh et al., 2002). Other studies have pointed out that negative attitudes might be rooted in a majority group’s cultural stereotypes about international students (Hanassab, 2006; Surtees, 2019), locals’ apprehension over being misunderstood (Myburgh et al., 2002; Surtees, 2019), and a host community’s bias against foreign accents and poor language proficiency (Kukatlapalli et al., 2020; Poyrazli and Lopez, 2007; Volet and Ang, 2012). Therefore, a comprehensive investigation of host community members’ attitudes and behaviors toward international students is needed to provide a holistic view of potential sources of negativity.

### 2.2 Integrated threat theory as a framework to understand prejudice

With its multidimensional approach to prejudice, Stephan and Stephan’s (2000) integrated threat theory offers a useful framework to determine potential sources of prejudice against international students in a host community. The framework is informed by Tajfel’s (1970) social identity theory which states that individuals create a positive self-image by emphasizing differences between members of the ingroup and outsiders belonging to various outgroups. Through the process of social differentiation, ingroup members perceive various types of threats from outgroup members (realistic threat, symbolic threat), experience intergroup anxiety, and express negative stereotypes, all of which can trigger prejudice.

While realistic threats concern the political or economic wellbeing of a group, symbolic threats encompass the group’s worldview captured through morals, values, and beliefs (Stephan and Stephan, 1996). For example, domestic students’ fear of receiving low grades when paired with international students in course projects or local residents’ belief that international students take away limited job resources from them can be described as realistic threats. Symbolic threats involve disagreements between international and domestic students regarding cultural values such as the perception shared by some students in the United Kingdom that their drinking culture is judged negatively by students from Muslim backgrounds (Harrison and Peacock, 2010). Intergroup anxiety illustrates the affective domain of intercultural contact, composed of feelings of apprehension, embarrassment, or rejection. When individuals expect their interaction to be unpleasant, they may see it as a personal threat due to the risk of losing face (Stephan, 2014; Stephan and Stephan, 1985), such as when domestic students feel reticent to interact with international students for fear of sounding racist or offensive (Harrison and Peacock, 2010). Lastly, negative stereotypes encompass people’s beliefs about outgroup members’ personal qualities, where international students might be labeled as bad at speaking a language, shy, or unsociable (e.g., Harrison and Peacock, 2010; Ruble and Zhang, 2013). From this perspective, negative stereotypes include preconceived ideas reflecting various degrees of misunderstanding of international students or their places of origin.

Harrison and Peacock’s (2010) qualitative investigation is among a handful of studies that have adopted the integrated threat theory to understand the treatment of international students (Mak et al., 2014; Spencer-Rodgers and McGovern, 2002). Through individual and focus group interviews with students in the United Kingdom, these researchers reported examples of various sources of bias against international students. For example, domestic students’ dissatisfaction with peer learning experiences and their concerns about receiving low grades were interpreted as realistic threats, whereas differences in behavioral norms (e.g., problems with punctuality, over-diligence) were attributed to symbolic threats. Students’ intergroup anxiety was linked to a lack of shared cultural reference points, language-related anxiety, and fear of committing social faux pas. Finally, negative stereotyping was evident in students’ overgeneralization of racial and ethnic descriptors (e.g., unfriendly, poor English skills) to entire ethnic groups, especially international students from China.

Although issues of language were subsumed in Harrison and Peacock’s (2010) study—and in the integrated threat theory more generally—under the comments pertaining to intergroup anxiety, language in and of itself could be a separate dimension contributing to prejudice. Dovchin’s (2020) interviews with international students in Australia, for instance, suggested that pronunciation mistakes lead to bullying and being “other-ed,” which may then cause students to experience lack of belonging, depression, and even suicidal thoughts. Having moved to New Zealand with her family at the age of three, one participant, for instance, mentioned that her good English was questioned (i.e., “How come you speak English this well?”) as she happens to be a Muslim woman wearing a hijab, which cued outgroup membership for some locals, revealing intricate links between social expectations and language attitudes (Kennedy et al., 2024). In a study focusing on domestic students’ willingness to interact with international students (Spencer-Rodgers and McGovern, 2002), responses to statements such as “I find it unpleasant to listen to foreign students who speak with a strong accent” and “I become impatient when listening to foreign students who speak English poorly” were among the strongest predictors of domestic students’ attitudes toward their international peers. Elsewhere, low-proficiency, accented speech has been shown to elicit unfavorable evaluations from course instructors (Jean-Francois, 2019), to trigger negative bias in employment contexts (Kukatlapalli et al., 2020), and to contribute to international students’ exclusion from group work (Haugh, 2016) and difficulty making friends with local students (CBIE, 2015). Thus, in addition to exploring various sources of prejudice against international students, this study also examines language-related attitudes (i.e., linguistic threat) as a separate dimension potentially contributing to prejudice.

### 2.3 Intergroup contact

Intergroup contact has been studied extensively in the literature on international students, with most studies suggesting that contact with locals tends to be problematic. Locals seem to avoid contact with international students, which is reflected in the low numbers of local friends reported by international students in many host communities such as the United States, the United Kingdom, and Australia (Gareis, 2012; Gbadamosi, 2018; Gresham and Clayton, 2011; Rajapaksa and Dundes, 2002). Despite its emphasis on multiculturalism (Government of Canada, 2018), Canada does not seem to fare better than other nations. According to an earlier report (CBIE, 2015), the percentage of Canadian friends for Middle Eastern and Asian students studying in Canada was low (28–44%), compared to the number of Canadian friends reported by American students in Canada (84%). In another Canadian study, only 10% of international students reported spending time with their local peers outside instruction, and the existing relationships between international and local students were superficial (Zhou and Zhang, 2014).

Even though intergroup contact involving international students is rare (e.g., Williams and Johnson, 2011), it provides a multitude of benefits for host communities and international students. For international students, apart from linguistic benefits such as improved sociolinguistic and pragmatic skills (Finn and Green, 2009; Kennedy Terry, 2022; Taguchi, 2011) and interactional competence (Masuda, 2011), positive social contact can also facilitate social adjustment (Hechanova-Alampay et al., 2002; Jean-Francois, 2019), reduce perceived discrimination, and alleviate negative emotional states and personal problems (Chataway and Berry, 1989; Ye, 2006). In addition, positive contact not only provides opportunities for intercultural communication but also encourages qualified international students to remain in their host community and contribute to its socioeconomic vitality (Netierman et al., 2021), particularly in countries with a slow population growth such as Canada (The World Bank, 2020), where immigration fuels population growth (Statistics Canada, 2023).

With respect to the role of intergroup contact in prejudice, there appears to be a complex reciprocal relationship between these constructs. For instance, institutionally supported intergroup contact, where individuals of equal social status interact in a cooperative manner toward a common goal, is more likely to reduce prejudice (Allport, 1954; Pettigrew, 1998). Similarly, frequency of contact mediates the relationship between perceived threat and attitudes (Corenblum and Stephan, 2001; Stephan et al., 2002) and sometimes predicts attitudes directly, where the more contact individuals have with a certain group (e.g., Muslims, cancer patients), the more positive attitudes they exhibit toward its members (Berrenberg et al., 2002; González et al., 2008). In other cases, prejudice can have either an equal or even stronger effect on the quantity of contact, where individuals with high levels of prejudice toward a group tend to avoid contact with its members (Binder et al., 2009). Finally, not only quantity but also quality of contact seems to play a role in prejudice (Allport, 1954), such that the quality of contact (e.g., cooperative vs. competitive, intimate vs. superficial) is often a better and more significant predictor of attitudes than its quantity (Binder et al., 2009; Mak et al., 2014; Stephan C. W. et al., 2000). Apart from attitudes, frequent and high quality (i.e., positive) contact may also reduce the level of perceived threat (Stephan and Stephan, 2000), which in turn reduces prejudice (Aberson, 2019). For instance, Stephan W. G. et al. (2000) found that the more frequent contact Americans had with Mexicans, the lower they scored on all types of threat. Therefore, in the present study, intergroup contact is operationalized through both quality and quantity to explore its relationship with host community members’ attitudes toward and perceived threat from international students.

### 2.4 The current study

As shown in prior research, international students often experience prejudice from members of the local community. However, apart from a few attempts (Harrison and Peacock, 2010; Mak et al., 2014; Spencer-Rodgers and McGovern, 2002), there is no comprehensive explanation as to potential sources of prejudice. Whereas some studies allude to the critical role of stereotypes, others highlight the incompatibility of social and cultural values between the two groups. Given that the research reviewed so far points to a crucial role of language in attitudes and behaviors toward international students, this study aims to contribute to this literature by incorporating linguistic threat as an additional explanatory variable, to supplement those already included within the integrated threat theory (realistic and symbolic threats, intergroup anxiety, negative stereotypes).

As much as it is crucial to investigate sources of host community members’ negative attitudes toward international students, it is perhaps even more important to determine whether these attitudes lead to prejudicial behaviors, as attitudes do not always reflect actions (Garrett, 2010). For example, local residents may harbor little prejudice against international students but may show no interest in communicating with them. Similarly, some prejudiced locals may be obligated to maintain contact with students because circumstances require them to do so, for instance, through work on common projects. Thus, to move beyond the realm of attitudes and to include behaviors, this study also aims to contribute to the literature on international students by exploring the association between host community members’ quantity and quality of contact with international students and their attitudes toward as well as perceived threat from these students. In the end, it is actions rather than attitudes that may have tangible consequences for students’ daily experience.

Last but not least, in their qualitative study, Harrison and Peacock (2010) provided domestic students’ perspectives regarding their challenges communicating with international students; however, they excluded members of the larger community residing off campus. Since prejudicial attitudes and discrimination likely extend beyond university campuses (Grayson, 2014; Lee and Rice, 2007), and in fact may be further amplified in those contexts (Hanassab, 2006), this study aims to provide a more comprehensive look at intergroup relations by recruiting participants—all representing members of the local community—from both student (i.e., domestic students) and non-student (i.e., local residents) populations.

The present study, which extends the work on prejudice within the integrated threat theory to include language as a potential source of bias against international students, was conducted in Montreal, Quebec. This context appears particularly suitable for investigating language as an additional source of prejudice in light of the importance of French to the ethnolinguistic vitality of francophones, Quebec’s majority ethnolinguistic group. Issues of language are central to the francophone identity, as illustrated by research on francophones’ attitudes toward French (Genesee and Holobow, 1989; Kircher, 2012; Lambert et al., 1960) and the Quebec government’s recent controversial legislation strengthening the status of French (National Assembly of Quebec, 2021). To elaborate, Bill 96 places the French language at the core of the Quebecois identity and charges the province with the task of ensuring the survival of “la francophonie” in North America. In Bill 96, the use of French is a critical condition for the integration of newcomers into Quebec’s society and a principal pathway for them to contribute to its future. Therefore, by virtue of their temporary, foreign-resident status, English-speaking international students might be perceived as unwilling to integrate and participate in Quebec’s society.

Given this background, if language issues contribute to prejudice against international students, these issues would most likely be salient in Quebec, where local residents (and especially non-student members of the local community) who hold strong beliefs about the preservation of French may be more inclined to perceive international students as a threat to the ethnolinguistic vitality of the francophone majority due to these students’ limited knowledge and use of French. Such concerns might be particularly relevant to international students enrolled in English-medium universities, given that Montreal hosts two such institutions that attract large cohorts of out-of-province and especially international students (World University Rankings, 2023), whose numbers nearly doubled in the past decade (Morasse, 2023). Therefore, the present study focuses on student and non-student members of the Montreal francophone community, exploring their attitudes toward and contact with international students at English-medium universities (i.e., students with presumed little or no proficiency in French). The study was guided by the following research questions:

1. With respect to student and non-student members of the Montreal francophone community, what are their attitudes toward, perceptions of threat from, and contact with international students?
2. Which variables (i.e., realistic threat, symbolic threat, intergroup anxiety, negative stereotypes, linguistic threat) account for the attitudes of the Montreal francophone community toward international students?
3. What is the association between Montreal francophone community members’ quality and quantity of contact with international students and their attitudes toward and perception of threat from them?

## 3 Method

### 3.1 Participants

Participants included 59 individuals (29 students, 30 non-students) who self-identified as francophone, listed French as one of their first languages (Leimgruber and Fernández-Mallat, 2021), and reported a minimum of 2 years of residence in Montreal to ensure their familiarity with its social and cultural landscape. Participants self-reported their listening, speaking, reading, and writing proficiency in English and French by using 100-point scales, where 0 corresponded to “not competent at all” while 100 meant “very competent,” and these scores were averaged to calculate the overall proficiency for each participant per language. Participants also reported the percentage (0–100%) of their daily language use (French, English, other) as well as their familiarity with L2-accented French and English, with 0 corresponding to “not at all” while 100 indicating “very much.”

Student participants (23 females, 5 males, and 1 non-binary), who were on average 26.9 years old (*SD =* 6.3, range = 18–46), pursued various academic degrees, including BA (14), MA (6), PhD (6), and other professional certificates (3), at English-medium universities in Montreal. Fourteen participants worked part-time (12 off campus, 2 on campus) during their studies. The majority of student participants (17) self-reported their ethnic identity as White; however, other ethnic groups such as East Asian, Black, Arab, West Asian, and Southeast Asian were also represented. Among the 25 Canada-born participants, the majority (24) were born in Quebec and one was born in Ontario. The remaining participants hailed from various parts of the world such as Algeria, China, Colombia, and Turkey. Their length of residence in Montreal was 20.6 years on average (*SD* = 8.5, range = 5.9–36). Based on self-reports, they were similarly proficient in both English (*M* = 95.2, *SD =* 6.07) and French (*M* = 95.2, *SD =* 7.4). However, as francophone students studying at English-medium universities, they reported greater daily use of English (*M* = 50.8, *SD =* 20.6) than French (*M* = 44.2, *SD =* 21.9) and greater familiarity with L2-accented English (*M* = 90.9, *SD =* 18.3) than French (*M* = 74.9, *SD =* 27.7).

Non-student participants (17 females, 13 males) had a mean age of 34.8 (*SD =* 7.5, range = 22–54) and were recruited from outside the university context to represent local francophone residents who were not pursuing an academic degree at the time of data collection. They were engaged in various professions such as a pastry chef, dance teacher, professional writer, receptionist, mental health counsel, and architect. While most (23) were White, other ethnicities such as East Asian, Black, Métis, and Southeast Asian were also represented among non-student participants. The majority (25) were born in Quebec, while four were born in France and one in Ontario, and all resided in Montreal for 21.5 years on average (*SD =* 11.3, range = 4.5–46.5). Non-student participants were more proficient in French (*M* = 96.0, *SD =* 6.2) than English (*M* = 86.2, *SD =* 12.5), and they reported using considerably more French (*M* = 76.8, *SD =* 19.8) than English (*M* = 20.5, *SD =* 18.0) on a daily basis. Finally, they were similarly familiar with L2-accented French (*M* = 80.8, *SD =* 29.3) and English (*M* = 82.3, *SD =* 19.1).

### 3.2 Materials

To determine which potential variables account for francophone host community members’ attitudes toward international students and to elicit the quality and quantity of intergroup contact between these groups, several measures of prejudice and intergroup contact were adapted from previous research. To help student and non-student participants reflect on their attitudes toward international students, a brief contextualizing statement was provided prior to presenting relevant questionnaire items (e.g., *Lorsque vous répondez aux questions ci-dessous, veuillez penser aux étudiant.e.s internationaux.les qui étudient dans les universités anglophones de Montréal, qui ont peu ou n’ont pas de connaissances en français et qui utilisent généralement l’anglais dans leurs activités quotidiennes sur le campus et en dehors du campus*. [As you respond to the items below, please think about international students enrolled in English-medium universities in Montreal with little or no French background and who generally use English in their day-to-day activities on and off campus]). To allow for comparability across measures, all questionnaire statements, except those targeting negative stereotypes (see below), were presented through 100-point sliding scales (with no numerical markers), where the two relevant endpoints were labelled negatively on the left (e.g., “totally disagree,” corresponding to 0) and positively on the right (e.g., “totally agree,” corresponding to 100) and the initial slider position was set in the middle. All questionnaire items were translated and presented to participants in French.

#### 3.2.1 Attitudes toward international students

Corenblum and Stephan’s (2001) evaluative/emotional reactions questionnaire was adopted to measure participants’ attitudes toward international students. The questionnaire elicited the degree to which host community members experience the following six positive emotions (approval, admiration, acceptance, affection, sympathy, warmth) and six negative emotions (dislike, superiority, hostility, disdain, hatred, rejection) when thinking about international students (not at all–very much).

#### 3.2.2 Sources of prejudice toward international students

Based on the integrated threat theory (Stephan and Stephan, 2000), there were four measures capturing various sources of prejudice (realistic threat, symbolic threat, intergroup anxiety, and negative stereotypes), along with a separate measure of linguistic threat which was specifically developed for this study. Realistic threat was assessed through seven agree–disagree statements adapted from previous research investigating domestic students’ attitudes toward international students in the United States (Spencer-Rodgers and McGovern, 2002) and New Zealand (Ward et al., 2005), with necessary adaptation to Quebec (see Data availability statement). For example, the statement “American colleges and universities are paying too much to finance the education of foreign students” was modified to read: “The government and universities in Montreal are paying too much to finance the education of these students.” Similarly, the statement “International students have a negative effect on the quality of New Zealand education” was altered as follows: “They decrease the quality of education in colleges and universities in Montreal.” To create comparable statements for student and non-student participants, minimal adaptations were introduced to reflect the lived reality of these two groups (e.g., “They take jobs away from local francophone students in Montreal [e.g., part-time employment as a barista off campus, teaching/research assistantships on campus]” for student participants and “They take jobs away from local francophone residents in Montreal [e.g., part-time employment as a shopping assistant, courier, food delivery person]” for non-student participants). Across all statements, the pronoun “they” referred to international students in English-medium universities, which was clear from the contextualizing statement.

Symbolic threat was captured through seven agree–disagree statements adopted from previous work (Spencer-Rodgers and McGovern, 2002; Ward et al., 2005), with word-level changes introduced to adapt each statement to the Quebec context (e.g., “Montreal is losing its Quebecois character because of the increasing number of these students”). All statements were identical for both student and non-student participants. Intergroup anxiety was measured following Corenblum and Stephan (2001), where participants were asked to indicate how they felt (not at all–very much) when interacting with international students by using 12 adjectives (apprehensive, friendly, uncertain, comfortable, worried, trusting, threatened, confident, awkward, safe, anxious, at ease). This measure was also identical for both student and non-student participants.

To measure negative stereotypes, a composite stereotype index was created following previous empirical work on prejudice and intergroup attitudes (Corenblum and Stephan, 2001; Esses et al., 1993) following Stephan and Stephan’s (1996) recommendation that researchers should avoid carrying out and interpreting correlations between prejudice and raw stereotype scores. To achieve this, 12 traits (calm, close-minded, clean, boastful, lazy, loud, passive, sociable, reliable, opportunist, considerate, hardworking), all relevant to international students, were selected from previous work on attitudes toward international students (e.g., Harrison and Peacock, 2010). Participants were first asked to indicate the percentage of international students who they thought may possess these traits (0–100%) and then to rate the favorableness (i.e., valence) of each trait on a 10-point scale, where −5 corresponded to “very unfavorable” and + 5 corresponded to “very favorable.” These scores were then used to create a composite stereotype/evaluation index (see Data Analysis).

The dimension of linguistic threat was captured through six statements eliciting francophones’ attitudes toward French in Quebec (e.g., “They must respect and accept Quebec government’s French-only policy in the public domain”), adapted from previous research (Gatbonton and Trofimovich, 2008) which targeted ethnic group affiliation of francophones in the same research context. There were two additional items tapping into the affective dimension of linguistic threat (e.g., “I feel tolerant toward them when they have poor skills speaking French”), following prior work on the integrated threat theory (Spencer-Rodgers and McGovern, 2002), for a total of eight items (totally agree–totally disagree). All statements for student and non-student participants were identical, except for two items which inquired about language preference on and off campus for students and inside and outside the work context for non-students.

#### 3.2.3 Intergroup contact

Following Spencer-Rodgers and McGovern (2002) and Ward et al. (2005), student participants’ frequency of contact with international students was assessed with respect to four different potential contexts of interaction (0 = “never,” 100 = “always”): working in a study group, sharing class notes, doing group assignments, and communicating during free time outside class (e.g., at coffee shops, restaurants, bars, etc.). These contexts were adapted for non-student participants, resulting in four different contexts of interaction: at work, in my neighborhood, when using public transportation, and off-work in the social domain (e.g., at coffee shops, restaurants, bars, etc.). The measure of contact quality consisted of six 100-point semantic differential scales adopted from Ward et al. (2005) asking participants how they would describe their interaction with international students: unequal–equal, involuntary–voluntary, superficial–intimate, unpleasant–pleasant, competitive–cooperative, and negative–positive. The scales were identical for both student and non-student participants.

### 3.3 Procedure

Because the study was conducted during the COVID-19 pandemic, which precluded easy access to participants, all data were collected through the online survey platform LimeSurvey,^1^ with several safeguards in place to ensure data quality (Nagle, 2019). For example, following prescreening, interested participants who met the eligibility criteria were assigned an individual, single-use token to access the survey. Moreover, participants were unable to skip any items or change their answers once submitted, and they were asked to advise the researcher of any problems they encountered while completing the survey. Participants were also encouraged to find a quiet space away from distractions to complete the survey. The time spent by participants on the survey was tracked, with the idea that responses from participants who completed the survey too fast (i.e., skipping through items) or too slowly (i.e., abandoning the survey for hours) would be eliminated if necessary.

First, participants were provided information about the purpose of the study and were asked to read and accept an online consent form. Next, if they chose to participate, they were informed about the structure of the survey and were given instructions about how to complete the questionnaires. Participants first completed the 12 items capturing their attitudes toward international students, which was followed by the statements targeting realistic threat (7 items), symbolic threat (7 items), intergroup anxiety (12 items), negative stereotypes (12 items), language attitudes (8 items), and intergroup contact (10 items). Finally, participants filled out a background questionnaire that elicited their demographic information, length of residence in Montreal, and knowledge of languages (see Data availability statement). Before the final submission of their responses, participants were provided with an open-ended textbox to note any issues they encountered in the survey or to leave any comments regarding the study. The entire survey was presented to and completed by participants in French, and participants received CA$20 as compensation for their time.

### 3.4 Data analysis

Before compiling the dataset, negatively coded items for language attitudes were reverse-scored so that, in all cases, higher scores corresponded to more positive attitudes. For realistic, symbolic, and linguistic threat as well as intergroup anxiety, positively coded items were also reverse-scored so that higher scores corresponded to greater threat or anxiety. All ratings (except negative stereotypes) were checked for item reliability by computing Cronbach’s alpha for each variable, separately for non-student participants (0.93 for attitudes, 0.77 for realistic threat, 0.81 for symbolic threat, 0.78 for linguistic threat, 0.93 for intergroup anxiety, 0.80 for negative stereotypes, 0.90 for contact quantity, and 0.83 for contact quality) and for student participants (0.92 for attitudes, 0.90 for realistic threat, 0.89 for symbolic threat, 0.80 for linguistic threat, 0.90 for intergroup anxiety, 0.70 for negative stereotypes after removing one item with a particularly low item-total correlation, 0.90 for contact quantity, and 0.89 for contact quality). These values were sufficiently high (*α* ≥ 0.70) and either comparable or in fact superior to those reported previously in similar work, particularly for stereotypes (e.g., *α* = 0.41–0.67 in Corenblum and Stephan, 2001; *α* = 0.44 in Stephan W. G. et al., 2000). Therefore, composite scores were computed for each variable by averaging the relevant responses per participant. For negative stereotypes, following Corenblum and Stephan (2001), a composite stereotype index was derived for each participant by multiplying each attributed percentage value (0–100%) by the relevant valence score (from −5 to 5) per trait, then computing the mean across the 12 traits. The resulting stereotype index ranged between −500 and 500.

Out of 59 participants, four reported difficulty with the stereotype measure which elicited participants’ subjective impression of the percentage of international students possessing a given character trait (e.g., hardworking, lazy). These participants commented about one stereotype item that sounded abstract for them (a different item for different participants). However, upon further examination of the data, these datapoints did not appear to be outliers; therefore, these four participants’ data were retained in all analyses. Participants completed the survey within about 35 min which was deemed reasonable based on pilot testing; therefore, all data were included in the dataset.

Because normality checks revealed non-normal distributions for multiple variables, robust statistics were performed through comparison of bootstrapped BCa 95% confidence intervals (CIs) for differences between group means, on the assumption that bootstrapped CIs are largely unaffected by the distribution of scores (Field, 2018), which makes these analyses robust to violations of normality and thus more preferable to traditional nonparametric tests (Larson-Hall, 2016, p. 74). Effect sizes were interpreted based on previous literature (Plonsky and Oswald, 2014), using Cohen’s *d* for between-group contrasts (0.40, 0.70, and 1.00) and *r* for correlation strength (0.25, 0.40, and 0.60), where each value designates small, medium, and large effects, respectively.

## 4 Results

As summarized in Table 1, both student and non-student participants generally responded positively toward international students (with mean scores above 70), where the overlapping bootstrapped 95% CIs suggested that both groups were similar in their responses. With the exception of linguistic threat, which was perceived higher by non-students than students (as shown through non-overlapping bootstrapped 95% CIs), both participant groups reported relatively low perceptions of threat in general. With respect to stereotypes, both the mean values (around 100) and bootstrapped 95% CIs suggested rather neutral perceptions of international students by both participant groups, considering that these scores could be as low as −500 and as high as +500. As for contact, quality seemed to be rated higher than quantity for both groups. However, compared to non-students, student participants (predictably) reported higher frequency and greater quality of contact with international students, again as shown through non-overlapping bootstrapped 95% CIs.

**Table 1:** Descriptive statistics for francophone participants’ overall attitudes and feelings of threat toward and contact with international students.

Measure	Students			Non-students		
	*M*	*SD*	95% CI	*M*	*SD*	95% CI
Attitudes	73.40	18.13	[67.15, 79.48]	74.33	17.08	[67.92, 80.34]
Realistic threat	14.46	18.28	[8.32, 21.59]	12.59	14.81	[8.32, 21.59]
Symbolic threat	20.31	20.86	[12.85, 28.21]	22.32	18.59	[16.11, 28.40]
Linguistic threat	35.12	19.24	[28.13, 42.34]	55.56	20.68	[48.20, 62.96]
Intergroup anxiety	21.11	15.30	[15.36, 26.85]	22.82	17.64	[17.55, 28.56]
Stereotype index	106.77	69.82	[83.82, 130.33]	99.23	73.34	[72.20, 125.83]
Contact quantity	66.37	25.34	[56.43, 75.71]	42.46	26.84	[33.13, 52.56]
Contact quality	74.00	19.63	[66.62, 81.00]	60.43	18.14	[53.86, 67.06]

All values are based on composite scores, with a minimum of 0 and a maximum 100, except for stereotype index, which ranges between −500 and +500.

To answer the first research question which targeted potential differences between student and non-student members of the Montreal francophone community with respect to their attitudes toward, perceptions of threat from, and contact with international students, the two groups were compared via independent-samples *t* tests (reported for reasons of transparency and completeness), focusing on a bootstrapped 95% CI for the mean difference as a measure of between-group difference. As shown in Table 2, non-students reported a greater level of perceived linguistic threat than students, and this difference appeared to be statistically significant as the 95% CI for the bootstrapped between-group difference did not include 0 and the estimated effect size was large (Plonsky and Oswald, 2014). The two participant groups were comparable with respect to other types of threat measures. However, in terms of the reported contact, non-students’ contact with international students was significantly less extensive in quality and quantity than that reported by students, again as shown through the 95% CIs for between-group differences that excluded 0, with estimated medium-size effects (Plonsky and Oswald, 2014).

**Table 2:** Comparison of student and non-student local francophones’ overall attitudes, perceived threat toward, and contact with international students.

Measure	*M_diff_*	95% CI	*t*	*p*	*d*
Attitudes	−0.93	[−9.67, 7.96]	−0.21	1.00	−0.05
Realistic threat	1.87	[−6.91, 10.79]	0.46	1.00	0.11
Symbolic threat	−2.01	[−12.37, 8.71]	−0.40	1.00	−0.10
Linguistic threat	−20.45	[−30.87, −10.20]	−3.99	< 0.001	−1.02
Intergroup anxiety	−1.70	[−10.49, 7.05]	0.41	1.00	−0.10
Stereotype index	7.54	[−26.96, 41.37]	0.42	1.00	0.11
Contact quantity	23.91	[10.19, 36.13]	3.62	< 0.001	0.92
Contact quality	13.57	[3.88, 22.37]	2.85	0.06	0.72

Although bootstrapped 95% CIs are used to infer significance, Bonferroni-corrected *p* values for independent-samples *t* tests, along with effect size estimates (Cohen’s *d*), are provided for transparency and completeness.

To answer the second research question focusing on which variables predict francophone host community members’ attitudes toward international students, two separate hierarchical regression analyses were conducted for student and non-student participants. The outcome variable was attitudes toward international students, whereas measures of realistic threat, symbolic threat, intergroup anxiety, negative stereotypes, and linguistic threat were entered as predictors. All predictor variables, except linguistic threat, were entered in Step 1 simultaneously (i.e., forced entry) following previous work (e.g., Berrenberg et al., 2002; Stephan et al., 1999a); linguistic threat was added in Step 2 to assess its unique contribution to attitudes, particularly in light of the important role that language plays in the present study’s sociolinguistic context. As can be seen in Table 3, initial checks revealed multicollinearity issues within each group. In particular, in the non-student group, there was a strong association between realistic and symbolic threat (*r* = 0.77); in the student group, realistic threat was highly correlated with both symbolic threat (*r* = 0.83) and intergroup anxiety (*r* = 0.78). Considering that similarly strong associations were observed in previous research (e.g., Berrenberg et al., 2002; Spencer-Rodgers and McGovern, 2002; Stephan C. W. et al., 2000; Stephan et al., 2002), suggesting a large overlap between these measures, realistic threat was excluded from all further analyses, which allowed for maintaining the largest set of distinct predictors in each regression model.

**Table 3:** Pearson correlations among all variables for non-student participants (above the diagonal) and for student participants (below the diagonal).

Variable	1	2	3	4	5	6
1 Attitudes	–	−0.80, [−0.92, −0.54]	−0.69, [−0.83, −0.49]	−0.40, [−0.67, 0.02]	−0.51. [−0.73, −0.28]	0.68, [0.47, 0.86]
2 Realistic threat	−0.61, [−0.73, −0.52]	–	0.77, [0.59, 0.92]	0.52, [0.24, 0.71]	0.36, [0.03, 0.71]	−0.58, [−0.77, −0.39]
3 Symbolic threat	−0.79, [−0.90, −0.67]	0.83, [0.69, 0.92]	–	0.62, [0.36, 0.78]	0.52, [0.23, 0.74]	−0.70, [−0.85, −0.43]
4 Linguistic threat	−0.59, [−0.84, −0.27]	0.70, [0.49, 0.88]	0.69, [0.40, 0.85]	–	0.36, [−0.11, 0.62]	−0.43, [−0.70, −0.03]
5 Intergroup anxiety	−0.76, [−0.86, −0.64]	0.70, [0.60, 0.89]	0.70, [0.45, 0.84]	0.68, [0.42, 0.84]	–	−0.66, [−0.85, −0.30]
6 Stereotypes	0.58, [0.26, 0.83]	−0.56, [−0.73, −0.40]	−0.57, [−0.75, −0.38]	−0.59, [−0.76, −0.38]	−0.68, [−0.83, −0.45]	–

Bootstrapped 95% CIs provided in square brackets.

After the removal of realistic threat, no correlations among the remaining predictors surpassed the benchmark of |0.70| (Field, 2018). For student participants, tests of multicollinearity revealed no tolerance values below 0.20 (0.37–0.52) and no VIF values above 10 (1.92–2.74). No residual values fell outside the ±2 benchmark, suggesting little bias, and no standardized residual value exceeded the ±3 value (−1.65–1.97), with Cook’s distance values all falling below 1.00 (0.00–0.55). For non-student participants, tests of multicollinearity revealed no tolerance values below 0.20 (0.36–0.62) and no VIF values above 10 (1.62–2.56). According to casewise diagnostics, there was only one case below −2 (−3.06), suggesting no significant issues (Field, 2018), and one standardized residual value below the ±3 threshold (−3.06); however, no Cook’s distance value exceeded 1.00 (0.00–0.56).

As shown in Table 4, the regression model for students demonstrated a good fit to the data in Step 1, *F*(3, 25) = 20.13, *p* < 0.001, with a total of 71% of variance explained (adjusted *R*^2^ = 0.67), suggesting good cross-validity of the model. Adding linguistic threat in Step 2 resulted only in a 2% change in model prediction and did not improve the model significantly (Δ*R^2^* = 0.004, *p* = 0.589); therefore, the best-fitting model included symbolic threat, intergroup anxiety, and stereotypes, but only symbolic threat, *t*(25) = −3.30, *p* = 0.003, and intergroup anxiety, *t*(25) = −2.23, *p* = 0.035, significantly predicted attitudes.

**Table 4:** Results of multiple regression analysis using threat variables as predictors of student francophones’ attitudes toward international students (*n* = 29).

Predictors	*R*	ΔR^2^	*B*	95% CI	SE B	*β*	*t*	*p*
Step 1								
Constant	0.84	0.71	91.25	[72.92, 108.43]	7.49		12.18	< 0.001
Symbolic threat			−0.44	[−0.78, −0.13]	0.13	−0.51	−3.30	0.003
Intergroup anxiety			−0.46	[−0.80, −0.12]	0.21	−0.39	−2.23	0.035
Stereotypes			0.01	[−0.07, 0.11]	0.04	0.03	0.17	0.863
Step 2								
Linguistic threat	0.84	0.004	0.09	[−0.28, 0.41]	0.16	0.09	0.55	0.589

As summarized in Table 5, the regression model for non-students also demonstrated a good fit to the data in Step 1, *F*(3, 26) = 10.54, *p* < 0.001, with a total of 55% of variance in attitudes explained (adjusted *R^2^* = 0.50), again suggesting good cross-validity of the model. Adding linguistic threat in Step 2 did not improve the model significantly (Δ*R^2^* = 0.001, *p* = 0.791); therefore, the best-fitting model included symbolic threat, intergroup anxiety, and stereotypes as predictors, but only symbolic threat significantly predicted attitudes, *t*(25) = −2.23, *p* = 0.03, *β* = −0.41.

**Table 5:** Results of multiple regression analysis using threat variables as predictors of non-student francophones’ attitudes toward international students (*n* = 30).

Predictors	*R*	ΔR^2^	*B*	95% CI	SE B	*β*	*t*	*p*
Step 1								
Constant	0.74	0.55	76.19	[55.83, 94.14]	9.69		7.87	< 0.001
Symbolic threat			−0.38	[−0.73, −0.06]	0.17	−0.41	−2.23	0.034
Intergroup anxiety			−0.06	[−0.53, 0.31]	0.17	−0.06	−0.36	0.722
Stereotypes			0.08	[−0.01, 0.19]	0.05	0.35	1.64	0.112
Step 2								
Linguistic threat	0.74	0.001	0.04	[−0.31, 0.33]	0.14	0.05	0.27	0.791

Finally, the third research question targeted the association between francophone participants’ quality and quantity of contact with international students and their attitudes toward them and their perceptions of threat from them. To address this question, Pearson correlation tests were run separately for student and non-student participants (see Table 6). With respect to attitudes, contact quantity showed no meaningful associations for either students (*r* = 0.20, *p* = 0.294) or non-students (*r* = 0.02, *p* = 0.931), with bootstrapped 95% CIs for each association crossing zero in each case. Contact quality, however, was significantly positively linked to attitudes both for students (*r* = 0.74, *p* < 0.001, 95% CI [0.45, 0.94]) and non-students (*r* = 0.54, *p* = 0.002, 95% CI [0.20, 0.79]), with medium-to-strong effects (Plonsky and Oswald, 2014). In all cases, greater contact quality with international students was associated with more positive attitudes toward them. In terms of the associations between contact measures and various threat variables, contact quality showed negative relationships with all threat variables in both groups, as shown through bootstrapped 95% CIs that exclude zero, with effect sizes ranging from medium to large. Contact quantity, on the other hand, yielded only one association with a reliable 95% CI, and only for student participants, namely, between contact quantity and intergroup anxiety (*r* = −0.30). Put differently, while greater contact quality was associated with reduced perception of threat from international students in all cases, greater contact quantity was associated with less intergroup anxiety for student participants only.

**Table 6:** Pearson correlations between contact quantity and quality and rated variables of attitudes and perceived threat.

Variable	Students (*n* = 29)		Non-students (*n* = 30)	
	Quantity	Quality	Quantity	Quality
Attitudes	0.20 [−0.16, 0.56]	0.74 [0.42, 0.94]	0.02 [−0.28, 0.30]	0.54 [0.21, 0.78]
Realistic threat	−0.10 [−0.42, 0.14]	−0.48 [−0.70, −0.32]	−0.14 [−0.46, 0.20]	−0.43 [−0.64, −0.21]
Symbolic threat	−0.23 [−0.51, 0.07]	−0.68 [−0.81, −0.55]	−0.20 [−0.51, 0.17]	−0.69 [−0.85, −0.41]
Linguistic threat	−0.17 [−0.44, 0.07]	−0.57 [−0.81, −0.31]	−0.28 [−0.58, 0.07]	−0.55 [−0.81, −0.13]
Intergroup anxiety	−0.30 [−0.61, −0.03]	−0.57 [−0.79, −0.33]	−0.19 [−0.46, 0.18]	−0.64 [−0.84, −0.34]
Stereotype index	0.37 [−0.03, 0.64]	0.59 [0.37, 0.80]	0.10 [−0.35, 0.44]	0.70 [0.45, 0.85]

## 5 Discussion

The present study investigated francophone host community members’ attitudes toward, perceptions of threat from, and contact with international students attending English-medium universities in Montreal. To provide a comprehensive picture of intergroup relations and contact, the study targeted francophone participants representing both student (on campus) and non-student (off campus) resident communities. Although there were no statistically significant differences between these two groups with respect to their attitudes toward and perceptions of all but one type of threat from international students (i.e., linguistic threat), their attitudes toward international students were predicted by a somewhat different combination of variables. Whereas symbolic threat was the sole significant predictor of attitudes for non-students, students’ attitudes were predicted by both symbolic threat and intergroup anxiety. Finally, compared to non-students, students reported higher frequency and greater quality of interaction with international students. Contact quality was associated positively with attitudes and negatively with all types of threat (except for stereotypes) for both participant groups. Contact quantity, on the other hand, was negatively linked to intergroup anxiety, and only for student participants.

### 5.1 Student versus non-student participants

In the present dataset, there were no significant differences between student and non-student participants in their attitudes toward international students, insofar as both groups of local francophones generally expressed positive views of international students. For student participants, this finding is in agreement with previous work, where domestic students generally expressed favorable attitudes toward international students on campus in various research contexts, including the United States, Australia, and New Zealand, despite reporting negative stereotypes about and perceived threat from international students (Quinton, 2019; Mak et al., 2014; Spencer-Rodgers, 2001; Spencer-Rodgers and McGovern, 2002; Ward et al., 2005). For non-student participants, this finding offers a positive perspective, considering that members of local communities often express only moderate approval of international students, for example, as documented in New Zealand (Ward et al., 2009), or in fact report negative sentiments toward international students (particularly from Asia and the Middle East), as reported in the United States (Hanassab, 2006). At least one reason for participants’ overall favorable attitudes likely stems from the sociolinguistic context of this study (Montreal), a multilingual and multicultural city with approximately 25% of its population representing individuals from over 100 different ethnic and cultural origins (Statistics Canada, 2021). Such diversity may have contributed to creating an atmosphere of open-mindedness in the city, which has been linked to less prejudicial attitudes (Williams and Johnson, 2011). Moreover, with four large public research universities and several other well-known institutions of higher education, Montreal is home to more than 175,000 students, around 18% of whom are international (La Chambre de commerce du Montréal métropolitain, 2016), rendering the population of students (international or otherwise) highly visible and therefore a common (i.e., “normal”) sight in the city. To sum up, the positive attitudes expressed by both student and non-student participants suggested a potentially welcoming environment for international students on and off campus.

In line with positive attitudes, both student and non-student francophone participants reported notably low levels of realistic and symbolic threat and intergroup anxiety as well as relatively neutral stereotypical beliefs, with no significant between-group differences. For realistic and symbolic threat, these findings are consistent with the idea that social groups which possess considerable political and socioeconomic power and stability are less likely to perceive realistic and symbolic threat from outgroup members or be overly impacted by intergroup anxiety (Stephan et al., 1999b). Indeed, there is strong evidence that Quebec’s francophone community feels secure in its socioeconomic status which has steadily risen over the past few decades based on variables such as mean income, the value of French in the job market, and the percentage of business ownership in the province (Albouy, 2008; Dean and Geloso, 2022; Gagnon et al., 2023; Vaillancourt et al., 2007). From a political standpoint, low levels of realistic and symbolic threat can also be attributed to the recently adopted legislation whose goal is to solidify the socioeconomic and cultural vitality of the francophone majority (Bourhis and Sioufi, 2017). For instance, passed in 2019, Bill 9 (An act to increase Quebec’s socioeconomic prosperity and adequately meet labor market needs through successful immigration integration) ensured that all newcomers to the province go through an extensive francization process with a strong emphasis on Quebecois values. Moreover, a steady decrease in anglophones in the province, coupled with an increase in francophone-owned businesses and in francophones’ purchasing power, may have also reinforced the socioeconomic status of Quebec’s French-speaking majority (Vaillancourt et al., 2007). Against this backdrop, it is not altogether surprising that francophones perceived little intergroup anxiety toward international students and engaged in little stereotyping about them (Mak et al., 2014; Ward et al., 2005), despite the steady increase in the number of international students (La Chambre de commerce du Montréal métropolitain, 2016). In essence, as members of the local francophone majority with considerable economic and sociopolitical power, both student and non-student participants in this study appeared to exhibit positive attitudes toward international students, to feel reasonably comfortable about interacting with them, and to have little reason to engage in stereotyping about them.

Compared to other types of threat, perceived linguistic threat from English-speaking international students was relatively high, especially for non-students for whom linguistic threat was greater than for students. Despite their strong socioeconomic status, the francophone majority appears to persist in experiencing linguistic vulnerability, likely due to the minority status of French against the backdrop of “anglonormativity” in the broader context of North America (Levesque, 2022), which can be described as “a system of structures, institutions, and beliefs that marks English as the norm” (Baril, 2017, p. 127). Therefore, despite the preventive measures put forth by the Quebec government inside the province such as Bill 96 (National Assembly of Quebec, 2021), francophone participants likely expected international students—as members of the anglonormative community from outside the province—to undervalue French. Indeed, among other linguistic threat items, the statement that concerned respecting and accepting the Quebec government’s French-only policy in the public domain elicited the strongest responses from both students (*M* = 59.0, *SD* = 33.0) and non-students (*M* = 77.1, *SD* = 23.1). Put differently, francophone participants may have felt threatened by the assumption that international students would disregard Quebec’s French-only language policy.

Even though all participants perceived a fair degree of linguistic threat from international students, non-students expressed a stronger degree of linguistic concern than students. These between-participant differences can be interpreted in several ways. First, positive interpersonal contact tends to reduce prejudice (Allport, 1954; Pettigrew, 1998) and to diminish perceived threat, which in turn attenuates prejudice (Aberson, 2019). For instance, Stephan W. G. et al. (2000) showed that, for their Mexican and American participants, greater quantity and quality of intercultural contact were associated with reduced symbolic and realist threat, and greater contact quantity was also linked to decreased intergroup anxiety (see also Mak et al., 2014). It is therefore unsurprising that student francophones, who reported having considerable contact (both in terms of its quantity and quality) with international students, perceived less linguistic threat from them. Second, student and non-student participants differed in their daily use of French and English, which may have played a role in their perception of linguistic threat. Students reported a fairly balanced use of English (50.8%) and French (44.2%), and there were correlations between their perceived linguistic threat and their English and French use, where greater perceived threat was associated with less daily use of English (*r* = −0.38, *p* = 0.04) and more daily use of French (*r* = 0.33, *p* = 0.08). Unlike students, non-students reported considerably greater daily use of French (76.8%) than English (20.5%), yet the correlations between daily language use and perceived linguistic threat followed a similar pattern and were in fact stronger in magnitude, where greater linguistic threat corresponded to less frequent use of English (*r* = −0.50, *p* = 0.005) and more frequent use of French (*r* = 0.45, *p* = 0.01). In essence, non-student francophones used French more frequently than student francophones; as a result, non-students may have had ample opportunity to observe the French skills of international students and other recent immigrants, even if these interactions were infrequent, which may have contributed to non-students’ greater perception of linguistic threat from international students. Third, student participants, who were generally younger than non-students (*d* = 1.14, 95% CI [0.58, 1.68]), may have also perceived less linguistic threat because younger generations of language speakers in Quebec appear to be less concerned about the sociolinguistic tension between English and French (Leimgruber and Fernández-Mallat, 2021). Finally, having chosen to study at an English-medium university, student participants may have held especially favorable attitudes toward English, for example, appreciating its value as a shared *lingua franca*, so they likely expected less threat from English compared to non-student members of the francophone community whose daily encounters with international students were not as frequent.

With regard to the quantity and quality of contact (see Table 2), non-students (as individuals who presumably only interact with international students off campus) predictably reported significantly lower frequency and quality of intergroup contact than students, who often have more opportunities for intergroup contact on campus, whose interactions with international students are frequently institutionally supported such as through intercultural activities (Allport, 1954), and whose status is more likely to be equal due to the shared student identity (Quinton, 2019). Nevertheless, non-student participants’ average value of contact quality was considerable (60.43 on a 100-point scale), and clearly above the scalar midpoint, which contrasts with previous reports of infrequent and most importantly superficial communication between members of the host community and international students in Canada (CBIE, 2015; Zhou and Zhang, 2014) and elsewhere (Harrison and Peacock, 2010). Similarly, for both participant groups, contact quality was always higher than quantity (see Table 2), and contact quality (rather than quantity) was always associated with diminished perceived threats and intergroup anxiety and with less negative stereotypical beliefs (see Table 6). This is a promising result, considering that it is the quality of contact rather than its quantity that enhances intergroup attitudes (Binder et al., 2009; Mak et al., 2014; Stephan C. W. et al., 2000). Overall, compared to non-student members of the local francophone community, francophone students attending English-medium universities have greater quantity and quality of contact with international students. However, irrespective of participant status, it was greater contact quality that corresponded to more favorable perceptions of international students and less threat perceived from them.

### 5.2 Predictors of attitudes toward international students

The second research question examined various predictors of francophone participants’ attitudes toward international students, and the regression models accounted for more variance in the attitudes from students (71%) than non-students (55%). In previous research comparing the predictive power of various aspects of the integrated threat theory, more variance in participants’ attitudes was predicted for members of a majority (dominant) group such as White people in North America than for members of a minority group such as Indigenous people in Canada or African Americans in the United States (Corenblum and Stephan, 2001; Stephan et al., 2002). Considering that both participant groups represented the francophone majority in the context of Montreal (Quebec), the reported differences in model prediction are most likely attributable to the nature of each group’s contact with international students. To illustrate, having greater frequency and quality of contact with international students, student participants may have formed richer and more refined opinions and beliefs about them, leading to more homogenous group-level attitudes. By contrast, non-student participants may have expressed variable perceptions of international students, based on their person-specific and often infrequent patterns of contact. To take another example, in a study of attitudes toward cancer and AIDS patients (Berrenberg et al., 2002), various subcomponents of the integrated threat theory accounted for more variance in participants’ attitudes toward AIDS patients (70%) than cancer patients (28%). Whereas AIDS has historically been stigmatized and narrowly associated with certain marginalized social groups and their lifestyles, cancer has been attributed to various causes, including hereditary and genetic reasons and life choices. In essence, perceptions are driven by people’s specific experiences, where more homogenous exposure experiences (and associated beliefs) result in stronger, more consistent group responses.

Even though both student and non-student participants reported rather low levels of threat in general, zero-order correlations demonstrated considerable associations between all threat variables and attitudes (see Table 3), ranging between −0.59 and −0.79 for students and between −0.40 and −0.80 for non-students, and symbolic threat was the only common significant predictor of attitudes for both participant groups (*cf.* Tables 4, 5). Thus, even in the absence of a strong perception of threat, participants who believed that Montreal is losing its Quebecois character due to the growing number of international students or that international students’ academic, social, and religious values are incompatible with those of Quebec harbored more negativity (e.g., disdain, superiority, rejection) toward international students. This finding likely reflects the awareness of many Quebec francophones that they are a cultural and linguistic minority in the broader Canadian context dominated by anglophones, which results in more concerns for francophones with respect to losing their distinct culture and language (Bouchard and Taylor, 2008). Between 2016 and 2021, the percentage of Canadians who reported English as their first official language increased from 74.8 to 75.5%, whereas the percentage for French decreased from 22.2 to 21.4% (Statistics Canada, 2022), which may feed into francophones’ desire to protect their values regarding religion and social life. In another context, for Mexican-born residents in the United States, symbolic threat similarly emerged as the only significant predictor of their attitudes toward local-born residents (Stephan C. W. et al., 2000; Stephan W. G. et al., 2000), likely because their culture and social values were perceived to be compromised in the dominant American culture around them.

The predictive power of symbolic threat in the present research context can also be attributed to Quebec’s long-standing emphasis on developing a distinct identity from the rest of Canada, particularly regarding such central issues as language, culture, education, politics, religion, and institutional organization (Secrétariat du Québec Aux Relations Canadiennes, 2017; Warren and Langlois, 2020). These initiatives may have amplified francophones’ need to protect Quebec’s unique character as a nation via shared values among its French-speaking residents, resulting in a heightened perception of symbolic threat from outgroups. For example, the importance of symbolic threat as a predictor of attitudes may stem from the juxtaposition of Quebec’s laïcité (secularism), which can be traced back to the Quiet Revolution in the 1960s and 1970s (Warren, 2020), with the religious, ethnic, and cultural diversity represented by international students. Indeed, across Canada, Quebec is among the least religious provinces (Angus Reid Institute, 2022), and its distinct political stance on religious accommodation such as the passing of Bill 21 (National Assembly of Quebec, 2019) highlights religion as a key marker of non-francophone identity, rendering it a prominent issue in the public’s eye. Last but not least, there was a strong intercorrelation between symbolic and realistic threat, which implies that the role of symbolic threat in francophone participants’ attitudes toward international students must be interpreted in conjunction with the role of realistic threat. Even though francophones constitute the demographic majority (around 90%) in most regions of Quebec (Statistics Canada, 2022) and the provincial government systematically safeguards their rights and freedoms, francophones’ minority status outside Quebec may still render them susceptible to various forms of perceived realistic threat from international students, for instance, as individuals who compete with local residents for university admission, student bursaries, or post-graduation employment.

In addition to symbolic threat, intergroup anxiety emerged as a negative predictor of attitudes for francophone students but not non-students. This difference potentially stems from contextual factors, in the sense that intergroup contact with international students may seem less likely and therefore less anxiety-inducing for non-student than for student francophones. Indeed, local students are predictably more likely to interact with international students, which may trigger anxiety in them, and when people feel anxious, their behaviors are more likely to be informed by perceived norms and stereotypes about outgroups. For instance, investigating intergroup attitudes and perceived threat between White and Indigenous Canadians, Corenblum and Stephan (2001) reported a significant negative correlation between intergroup attitudes and anxiety for both groups. In a study of Black and White American students’ attitudes toward one another (Stephan et al., 2002), intergroup anxiety again emerged as a strong negative predictor of attitudes. Similarly, in an interview study (Harrison and Peacock, 2010), local-born students in the United Kingdom felt that intergroup contact generated misunderstandings and embarrassment stemming from cultural and linguistic differences between them and international students. Against this backdrop, intergroup anxiety appears to be associated not only with attitudes but also with behavior. Just as international students who feel particularly anxious not only express negative attitudes toward local students but also actively avoid interaction with them (Fritz et al., 2008; Harrison and Peacock, 2010; Kormos et al., 2014; Williams and Johnson, 2011), as the present findings suggest, local students similarly experience intercultural anxiety and associate it with negative views about international students. More importantly, for local residents and students in particular, increased intercultural anxiety is associated with decreased quantity and quality of contact with international students (see Table 6), which is a novel finding in the Canadian context.

Stereotypes failed to emerge as a significant predictor of both student and non-student francophones’ attitudes toward international students. With regard to student francophones, as younger individuals, they might be more concerned about self-relevant threats than group-level issues, including stereotypes, compared to non-student francophones, who are relatively older working professionals (Aberson and Gaffney, 2008). As for non-student francophones, less reliance on stereotypes in terms of intergroup attitudes can be attributed to their reported contact patterns with international students. Despite reporting lower frequency and quality of contact with international students compared to student francophones, non-student francophones still reported close to average contact quantity as well as above-average contact quality with international students. Thus, positive contact may have helped non-student francophones question common stereotypes about international students and view them as a heterogenous group of individuals instead of an undifferentiated group with identical traits.

Last but not least, one of the goals of this study was to examine the predictive validity of linguistic threat for francophone participants’ attitudes toward international students. In fact, linguistic threat was expected to have a unique contribution to attitudes, in light of recent legislation aiming to strengthen the status of French such as Bill 96 (National Assembly of Quebec, 2021) and increased support for French inside and outside Quebec (Bouchard, 2023; Kircher, 2012, 2014; Leimgruber and Fernández-Mallat, 2021). Although linguistic threat was negatively associated with attitudes for both students and non-students (see Table 3), this variable failed to account for unique variance in their attitudes after accounting for other threat variables. Instead of suggesting that language does not play a role in francophones’ attitudes toward international students, this finding most likely reflects how language has been intertwined with the values and beliefs systems of francophone Quebecers and how language cannot be easily separated from other types of threat (e.g., symbolic threat). That is, for local francophones, French has both a linguistic and a symbolic value, as it lies at the heart of Quebec francophone identity (Secrétariat du Québec Aux Relations Canadiennes, 2017, p. 14; Warren and Langlois, 2020) and critically functions as a common, unifying element across Quebec’s population (Warren and Oakes, 2011). The current findings likely reflect this reality, as shown by strong associations between linguistic and symbolic threats for both students (*r* = 0.69) and non-students (*r* = 0.62). Thus, in certain contexts such as Quebec where language is a key aspect of social identity, linguistic and symbolic threats are inherently intertwined.

### 5.3 The contact–attitudes link

With respect to the third research question, which explored the link between participants’ contact with international students and their attitudes toward them, contact quality rather than quantity showed a significant positive relationship for both students and non-students. Participants who reported greater contact quality with international students (e.g., evaluating it through such descriptors as intimate, positive, and cooperative) expressed more favorable attitudes toward them, demonstrating more acceptance, affection, and approval of international students. This result is consistent with the idea that high-quality (i.e., positive) contact enhances attitudes between members of different groups (Allport, 1954; Pettigrew and Tropp, 2006) and that the link between attitudes and contact is stronger for contact quality than quantity (Berrenberg et al., 2002; Binder et al., 2009; Mak et al., 2014; Stephan C. W. et al., 2000). For instance, in a study of attitudes and contact between Australian-born and international students, contact quality was the strongest predictor of intergroup attitudes, whereas contact quantity did not produce any significant relationship (Mak et al., 2014). Therefore, the present findings yet again emphasize the importance of quality over quantity with respect to the contact–attitudes link between host community members and international students in the Canadian context.

Contact quality was also significantly associated with all perceived threats, which is in line with previous research in Canada. For instance, greater contact quality was associated with reduced realistic threat and intergroup anxiety for Indigenous Canadians, whereas greater contact quality was similarly linked to less realistic and symbolic threat, decreased intergroup anxiety, and less negative stereotyping for White Canadians (Corenblum and Stephan, 2001). Investigating attitudes and contact between two religious groups in Northern Ireland, Tausch et al. (2007) similarly found a significant negative association, where greater perceived threat (in the form of symbolic and realistic threat and intergroup anxiety) was associated with less contact quality. Therefore, the present study not only confirms the link between contact quality and various perceived threat variables (Islam and Hewstone, 1993; Mak et al., 2014; Stephan W. G. et al., 2000) but also extends this work by demonstrating a similar association between contact quality and linguistic threat (Mak et al., 2014). Considering the central role that language plays in distorting one’s perception of others as well as in expressing, perpetuating, or revealing prejudicial attitudes (Collins and Clément, 2012), this finding is unsurprising. For both student and non-student francophones, low quality contact with international students was linked with heightened linguistic threat. To elaborate, when contact with international students was perceived as less favorable (e.g., superficial, competitive), international students were considered to pose greater threat to the status of French in Quebec and to be less willing to accommodate to French during intergroup encounters. A major takeaway from this finding is that attempts to reduce perceived threat from international students, particularly linguistic threat, should include efforts to enhance the quality of contact between international students and francophone host community members.

Even though a recent meta-analysis of the contact–prejudice relationship suggests that contact quantity alone, such as more frequent interactions between ingroup and outgroup members, may have the power to reduce intergroup prejudice (Aberson, 2019), in the present study, contact quantity revealed no association with participants’ attitudes. A diminished role of contact quantity in attitudes can be attributed to Montreal’s sociocultural and sociolinguistic diversity, where it might be difficult to differentiate international students from similarly diverse local residents, given that the saliency of a person’s group identity plays a major role in the contact–prejudice link (Hewstone, 2000). In other words, francophone participants may not have been fully aware of the frequency of their interactions with international students because the city’s local population is highly diverse. Unless a speaker discloses their status, distinguishing international students from recent immigrants or members of local-born minority communities might be a difficult if not impossible task. In fact, the only association involving contact quantity was a weak negative correlation with intergroup anxiety for student participants, implying that international students have a salient group identity on campus. International students are also likely to disclose their status to their classmates, with whom they presumably interact regularly, and in North American academic contexts, international students’ status is clearly demarcated institutionally, such as through tuition differentials, availability of funding, and required coursework, all of which makes international students recognizable to their peers. For francophone students, then, increased quantity of communication with international students was associated with decreased interpersonal anxiety rather than improved attitudes (Stephan et al., 1999b), which were already quite positive. Just as for Muslims and Hindus, who both represent identifiable and large groups in India (Tausch et al., 2009), for francophone and international students in English-medium universities in Montreal, greater quantity of contact was associated with decreased intergroup anxiety, irrespective of the attitudes they might hold. Thus, whereas the quality of interpersonal contact might have strongest links to attitudes, contact quantity is additionally implicated in reduced intergroup anxiety, which is a positive finding.

### 5.4 Limitations and future research

The present study is not without limitations. First, one key limitation is the relatively small sample size which limits the generalizability of the findings. Participant recruitment was particularly challenging during and immediately after the COVID-19 pandemic. Therefore, future research should aim to recruit a larger number of participants when and if possible. A further concern regarding participants is that, to avoid the impact of generational differences on intergroup attitudes, non-students were recruited to match students in age, so off-campus participants represented a relatively young cohort of francophones, with a mean age of about 35. Moreover, both participant groups had above-average English skills, which may have led to more acceptance (with regard to linguistic threat, in particular) of English-speaking international students. Therefore, in future work, it would be important to target older individuals with less proficiency in English, to achieve a better representation of Quebec’s society. Similarly, all participants had resided a minimum of 2 years in Montreal, which may have been insufficient for them to create and express attitudes toward international students, or to develop and act on potential contact opportunities with them. Clearly, a time-sensitive perspective on attitudes is needed to understand how local residents develop and change their reactions to newcomers, including international students. Second, despite reaching high levels in both participant groups, linguistic threat did not emerge as a significant predictor of attitudes toward international students. While this can be attributed to the intrinsically complex relationship between linguistic and symbolic threat in Quebec, it is also possible that the operationalization of linguistic threat in the present study may have failed to capture the more nuanced aspects of language in terms of attitudes, particularly those that are separate from its symbolic elements. Thus, future research could further enhance this construct through more refined measurements.

Third, due to a strong association between realistic and symbolic threat (Berrenberg et al., 2002; Spencer-Rodgers and McGovern, 2002; Stephan C. W. et al., 2000; Stephan et al., 2002), which led to initial multicollinearity issues in both participant groups, realistic threat was excluded from the regression analyses, thus allowing for the largest set of distinct predictors to be included in each model. Therefore, the importance of symbolic threat and its association with realistic threat in predicting attitudes should be interpreted cautiously. One way in which future research could sidestep this limitation would be by recruiting more participants to attain more robust regression models and by developing (rather than adapting) measures of threat variables that are specific to the Quebec context. Fourth, unlike symbolic threat, which was a significant predictor of attitudes for both student and non-student francophones, linguistic threat did not emerge as a significant predictor, despite initial expectations. Considering the sociolinguistic context of Quebec, this finding was attributed to the complex role language plays in the francophone identity. To further confirm this interpretation, further research is warranted in similarly complex sociolinguistic contexts such as Catalonia (i.e., Spanish vs. Catalan) or Wales (i.e., English vs. Welsh), where language is a distinct marker of one’s social identity and a subject of sociopolitical debate. Finally, as the present findings are informed by quantitative analyses only, future qualitative work could provide an in-depth, complementary view of intergroup attitudes and contact between international students and the host community, for instance, through focus group interviews with students from different sociocultural and linguistic backgrounds.

### 5.5 Implications

Considering that only 36–40% of international students in Canada remain in their host provinces after graduation (Choi et al., 2021), these findings can guide institutional and governmental efforts in facilitating intergroup relations and contact between host community members and international students both on and off campus, with the goal of retaining international talent after graduation. The present findings that highlight the significance of symbolic threat to the host community’s attitudes are of particular importance, because it is largely sociocultural variables, including issues of culture and values, that appear to impact international students’ decision to leave or stay upon graduation (Esses et al., 2018). In this regard, institutions of higher education could collaborate with such local organizations as municipal administrations, borough councils, and non-governmental entities to create promotional billboards, videos, and flyers, or organize practical workshops, panels, and Q&A sessions which directly involve both international students and host community members and dispel potential myths (e.g., various forms of perceived symbolic and realistic threats) through guided and supported interactions. Given a negative association between contact quality and symbolic threat, any such interaction should ensure cooperation and equality between the host community and international students and should be conducive to deeper-level intergroup communication. For instance, the above-mentioned local organizations could directly involve international student voices and encourage collaborative practices between the two groups. Opportunities such as these will help host community members to better understand the lived realities of international students and will encourage international students to forge stronger social and professional bonds with host community members, leading to healthy, symbiotic relationships between international students and the host community.

## 6 Conclusion

This study extended prior research on intergroup attitudes and contact between host community members and international students in the sociolinguistically vibrant context of Montreal. Even though both participant groups expressed similarly positive attitudes toward and little perceived threat from international students (except for linguistic threat), symbolic threat significantly accounted for both groups’ attitudes, and intergroup anxiety additionally accounted for student participants’ attitudes. Contrary to expectations, linguistic threat did not emerge as a significant predictor of francophones’ attitudes toward international students, which was attributed to the key symbolic role that French plays in Quebec’s social identity and values. Compared to non-student francophones, student francophones also reported more frequency and greater quality of contact with international students. Last but not least, for both participant groups, contact quality appeared to have a stronger relationship with perceptions about international students than contact quantity, where greater quality of contact was associated with reduced perception of threat, more favorable attitudes, decreased interpersonal anxiety, and less negative stereotyping.

## Data Availability

All data and materials for this study are publicly available via the Open Science Framework: https://osf.io/8x27w.

## Footnotes

^1^ https://www.limesurvey.org
